# Stretching Your Energetic Budget: How Tendon Compliance Affects the Metabolic Cost of Running

**DOI:** 10.1371/journal.pone.0150378

**Published:** 2016-03-01

**Authors:** Thomas K. Uchida, Jennifer L. Hicks, Christopher L. Dembia, Scott L. Delp

**Affiliations:** 1 Department of Bioengineering, Stanford University, Stanford, California, United States of America; 2 Department of Mechanical Engineering, Stanford University, Stanford, California, United States of America; 3 Department of Orthopaedic Surgery, Stanford University, Stanford, California, United States of America; Delft University of Technology (TUDelft), NETHERLANDS

## Abstract

Muscles attach to bones via tendons that stretch and recoil, affecting muscle force generation and metabolic energy consumption. In this study, we investigated the effect of tendon compliance on the metabolic cost of running using a full-body musculoskeletal model with a detailed model of muscle energetics. We performed muscle-driven simulations of running at 2–5 m/s with tendon force–strain curves that produced between 1 and 10% strain when the muscles were developing maximum isometric force. We computed the average metabolic power consumed by each muscle when running at each speed and with each tendon compliance. Average whole-body metabolic power consumption increased as running speed increased, regardless of tendon compliance, and was lowest at each speed when tendon strain reached 2–3% as muscles were developing maximum isometric force. When running at 2 m/s, the soleus muscle consumed less metabolic power at high tendon compliance because the strain of the tendon allowed the muscle fibers to operate nearly isometrically during stance. In contrast, the medial and lateral gastrocnemii consumed less metabolic power at low tendon compliance because less compliant tendons allowed the muscle fibers to operate closer to their optimal lengths during stance. The software and simulations used in this study are freely available at simtk.org and enable examination of muscle energetics with unprecedented detail.

## Introduction

The complexity of the human body is a byproduct of millions of years of evolution. Energetic requirements have increased over the course of human evolution as the hominid brain increased in size and capability [1]. We are driven to maximize energy intake and minimize energy use. It is thought that our ancestors developed hunting [2] and scavenging [3] strategies to obtain calories, and may have first adopted bipedalism in an arboreal environment to harvest fruits efficiently [4]. Humans have evolved mechanisms that decrease caloric expenditure during locomotion, adopting a bipedal gait and naturally selecting walking speeds [5, 6] and running stride lengths [7, 8] that are most energetically economical. The drive to minimize the energy required for locomotion is also evident in the physiology of our musculoskeletal system.

The structure and dynamics of the ankle plantarflexor muscles have evolved in a direction that makes locomotion efficient. The triceps surae muscle group has evolved to allow storage (during the braking phase) and release (during the push-off phase) of elastic strain energy in the calcaneal (Achilles) tendon [9]. Cavagna et al. [10] estimated that the positive mechanical energy returned by all the tendons in the lower extremity accounts for half the total positive mechanical work performed during running. Although this estimate is almost certainly too high [11], tendon compliance is nevertheless believed to play a critical role in determining energy consumption. The compliance of the calcaneal tendon in particular reduces the shortening velocity of the soleus and gastrocnemius muscle fibers, allowing them to operate closer to the length and velocity at which they can generate maximal force [5, 12–16]. These mechanisms substantially reduce the metabolic energy consumed by the plantarflexors during both walking and running [13, 17–19].

Several studies have investigated the sensitivity of triceps surae energy consumption to calcaneal tendon compliance and running speed. Lichtwark and Wilson [20] used a model of an isolated medial gastrocnemius musculotendon actuator during running at 2.8 m/s, and found that the simulated muscle was most efficient when the calcaneal tendon was at its physiological stiffness of 180 N/mm. Lai et al. [21] found that the elastic strain energy of the calcaneal tendon accounts for a greater proportion of positive musculotendon work than the work done by the soleus and gastrocnemius muscle fibers, and that this proportion increases as running speed increases from 2.1 to 9.0 m/s. These findings corroborate the results of Cavagna et al. [22], who reported over four decades earlier that tendons contribute a greater proportion of power at higher running speeds. Simulations performed by Dorn et al. [23] suggest that the plantarflexors are primarily responsible for increasing running speed up to 7.0 m/s (by increasing stride length), while the hip muscles play a more substantial role above this speed (by increasing stride frequency). These findings suggest that the economy of running is greatly affected by the dynamics of the calcaneal tendon, and that the relative importance of this tendon to overall running economy varies with speed. Many studies have examined the mechanics of running using performance metrics such as muscle force generation capacity [14, 16, 24] and mechanical work [13, 18, 21, 22, 25]; however, these metrics ignore heat generation, the consideration of which is essential for determining overall running economy. No studies have used a detailed whole-body musculoskeletal model to investigate the effect of tendon compliance of all the lower limb muscles on the metabolic cost of running, and thus the energetic effects of compliance of the other tendons in the lower limb remain unknown.

The physical properties of tendons have been measured by many researchers; the stress–strain relationship is the property most pertinent to the present study. At low strain, the slope of the stress–strain curve increases with strain as the collagen fibers straighten; at high strain, the slope of this curve is approximately constant until rupture [26]. In humans, tendon strain at failure appears to vary with age, ranging from 14–18% in infants to 10–12.5% in adults [27]; however, temperature, exercise, steroid use, diabetes, and hemodialysis can also affect the mechanical properties of tendons [28]. Furthermore, a number of factors affect the experiments performed on tendons, including species, age, fiber organization in the specimen, antemortem donor history, tissue storage methods, gripping techniques, and strain rate [26]. Consequently, the strain at maximum stress for both whole tendons and isolated fiber bundles has been reported to vary over a wide range, from 5 to 20% [29]. Magnusson et al. [30] were the first to measure the stress–strain characteristics of the human triceps surae tendon and aponeurosis in vivo, and reported tendon strains of 4.4±0.5% and 5.6±0.4% based on the displacement of the aponeurosis at its proximal and distal ends (i.e., at the distal part of the medial gastrocnemius head and the distal part of the soleus muscle) during maximal voluntary contractions. Maganaris and Paul [31] studied the human gastrocnemius tendon in vivo and reported similar strains of 4.9±1% during maximal voluntary contractions.

In the present study, we investigated the effect of tendon compliance on the metabolic cost of running using a full-body musculoskeletal model with a detailed model of muscle energetics. We performed muscle-driven simulations of running at several speeds and tendon compliances, and computed the average metabolic power consumed by each muscle. We used modeling and simulation to gain insight into the energy consumed by individual muscles throughout the gait cycle, a study that would be impossible to perform experimentally. We compared trends observed in muscle activations, metabolic power, and fiber mechanical power over a broad range of tendon compliances and at four running speeds, where tendon compliance was parameterized by the tendon strain as muscles were developing maximum isometric force (*F*_max_). We explored three hypotheses: (i) whole-body metabolic power consumption is a convex function of tendon compliance, reaching its lowest value when tendon strain is approximately 3–4% at *F*_max_, the range of tendon stretch suggested by Zajac [32]; (ii) for very low and very high tendon compliances, the muscles crossing the ankle experience a greater increase in metabolic power than those crossing the knee or hip; and (iii) metrics based solely on muscle activations or positive fiber mechanical power exhibit trends that are different from the predictions of a detailed muscle energetics model.

## Methods

Simulations were generated for 10 male long-distance runners using the motion and force data collected by Hamner and Delp [33]. The Stanford University Institutional Review Board approved the experimental protocol and subjects provided informed written consent. Seven subjects were rearfoot strikers at all running speeds; three were forefoot strikers whose data were excluded from analyses of muscle fiber lengths to facilitate comparison of fiber kinematics for different tendon compliances. The Computed Muscle Control (CMC) tool in OpenSim 3.2 [34, 35] was used to generate muscle-driven simulations of running at 2, 3, 4, and 5 m/s for each subject. CMC solves the muscle redundancy problem by minimizing the sum of squared muscle activations. We used a three-dimensional musculoskeletal model with 29 degrees of freedom, 92 lower extremity and torso muscles, and arms driven by torque actuators [36]. Among the quantities computed by the CMC tool are the activation, fiber length, and fiber velocity of each muscle over time. The CMC simulation results were used as inputs to a modified version of the muscle energetics model proposed by Umberger et al. [37]; our modifications are described in the Muscle Energetics Model section, below. Parameters for this model include the slow- and fast-twitch fiber composition of each muscle, which we obtained from Johnson et al. [38], Garrett et al. [39], and Alway [40]. Another key parameter in the muscle energetics model is muscle mass, *m*, which we calculated for each muscle from its physiological cross-sectional area and optimal fiber length, the length at which muscle fibers generate maximum isometric force:

$$\begin{array}{c} m=\rho \frac{F_{\text{max}}}{\sigma }\ell_{\text{opt}}, \end{array}$$ (1)

where *ρ* = 1059.7 kg/m^3^ is the density of mammalian muscle [37], and *F*_max_, *σ*, and ℓ_opt_ are the maximum isometric force, specific tension, and optimal fiber length of the muscle. We used the same specific tensions in Eq (1) as were used by Hamner and Delp [33] to compute *F*_max_ from measured physiological cross-sectional areas.

**Fig 1:**
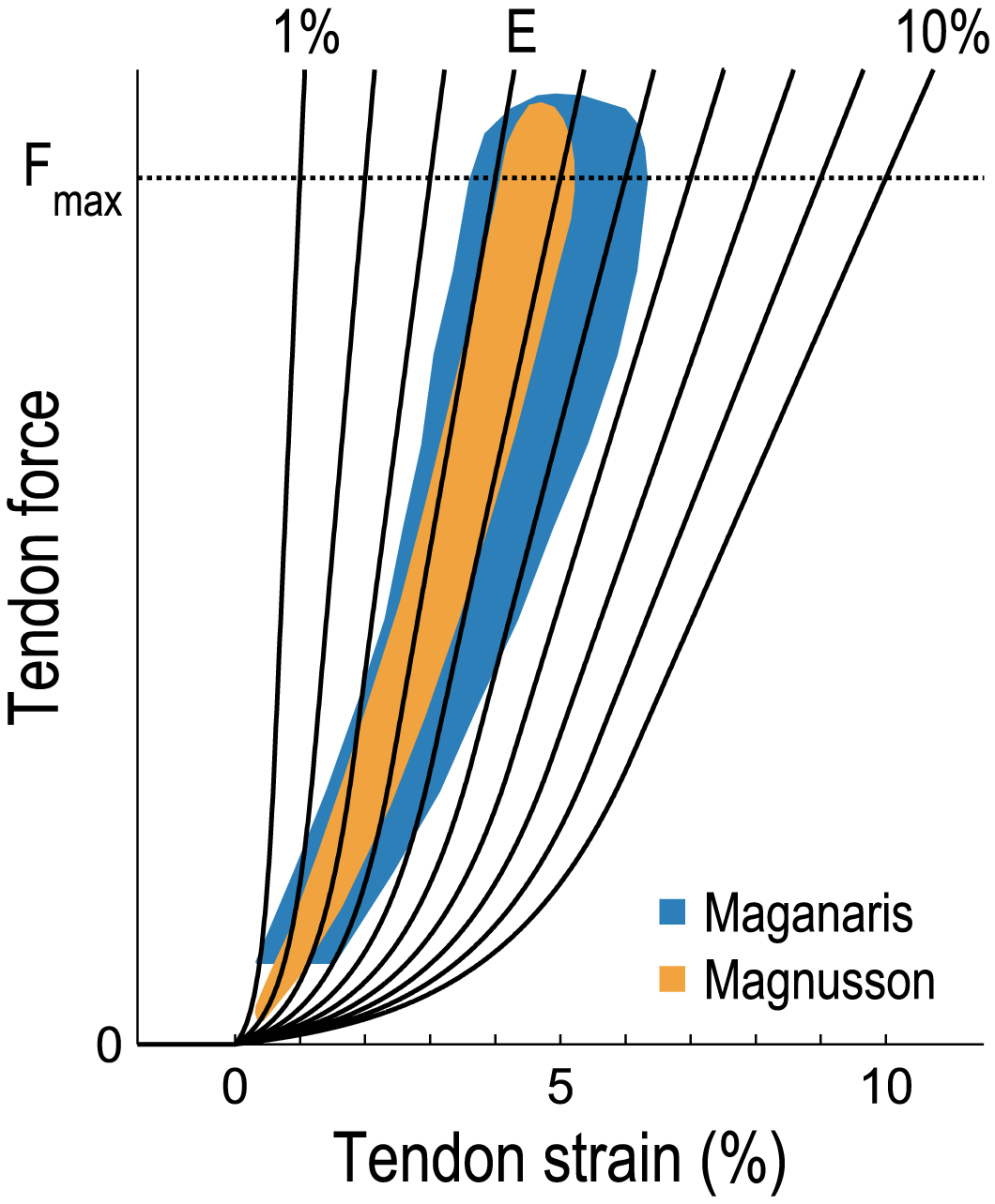
Tendon force–strain relationship measured experimentally and used in our simulations. The model curves (solid black lines) produced tendon strains of 1–10% when the muscles were developing maximum isometric force (*F*_max_). The curve labeled “E” is one physiologically plausible curve based on the experimental data reported by Maganaris and Paul [31] and Magnusson et al. [30] (shaded regions).

We simulated three running gait cycles for each subject at each speed, using the methods reported by Hamner and Delp [33]. The compliance of the tendon in each musculotendon actuator was characterized by the force–strain curve shown in Fig 1. The curve was parameterized by the strain at *F*_max_, where a greater strain at *F*_max_ corresponds to a more compliant tendon. We first used tendon force–strain curves that produce 4% strain at *F*_max_, which lies within the experimental range shown in Fig 1. We then repeated each simulation using tendon force–strain curves that produce between 1 and 10% strain at *F*_max_. The tendon force–strain curves associated with all musculotendon actuators were adjusted simultaneously and uniformly while the joint kinematics tracked by CMC were held fixed.

### Muscle Energetics Model

Our model of muscle energetics differed from that proposed by Umberger et al. [37] in two respects. First, we used an orderly recruitment model similar to that described by Bhargava et al. [41] to determine the ratio of slow- to fast-twitch fibers that were excited at each instant of the simulation:

$$\begin{array}{c} u_{\text{slow}}\left(t\right)=\sin(\frac{\pi }{2}u\left(t\right)), \end{array}$$ (2)

$$\begin{array}{c} u_{\text{fast}}\left(t\right)=1-\cos(\frac{\pi }{2}u\left(t\right)), \end{array}$$ (3)

$$\begin{array}{c} f_{\text{slow}}^{\text{rec}}\left(t\right)=\{\begin{array}{cc} 1, & \;\text{if}\;u(t)=0\\ \frac{f_{\text{slow}}u_{\text{slow}}\left(t\right)}{f_{\text{slow}}u_{\text{slow}}\left(t\right)+(1-f_{\text{slow}})u_{\text{fast}}\left(t\right)}, & \;\text{otherwise}, \end{array} \end{array}$$ (4)

where *u*(*t*) is the muscle excitation computed by CMC, *u*_slow_(*t*) and *u*_fast_(*t*) are the excitation levels of the slow- and fast-twitch fibers, *f*_slow_ is the fraction of fibers comprising the muscle that are slow-twitch fibers, and $f_{\text{slow}}^{\text{rec}}\left(t\right)$ is the fraction of recruited fibers that are slow-twitch fibers. When the muscle excitation *u*(*t*) is low, the fibers that are recruited are primarily slow-twitch fibers [42, 43], which consume less metabolic power than fast-twitch fibers. As excitation increases, the proportion of recruited fibers that are slow-twitch fibers decreases until, at maximum excitation, this proportion is equal to the proportion of slow-twitch fibers constituting the muscle (i.e., all fibers in the muscle are recruited and $f_{\text{slow}}^{\text{rec}}\left(t\right)=f_{\text{slow}}$). We used the fraction of recruited fibers that are slow-twitch fibers ($f_{\text{slow}}^{\text{rec}}\left(t\right)$) in place of the fraction of slow-twitch fibers comprising the muscle (*f*_slow_) in our implementation of the muscle energetics model proposed by Umberger et al. [37].

The second distinction addressed the treatment of negative mechanical work. The first law of thermodynamics suggests that negative mechanical work should be included in the energetics model, as is evident upon considering the fibers to be within a control volume—that is, upon drawing an imaginary boundary around the fibers and analyzing the flow of all forms of energy across this boundary. The original model proposed by Umberger et al. [37] included negative mechanical work, but the revised version [44] did not. The handling of negative mechanical work is a point of disagreement among muscle energetics models, and accounts for a substantial amount of the discrepancy between their predictions [45]. We chose to include negative mechanical work, and also used the equation for the lengthening heat rate coefficient from Umberger et al. [37] (this equation was changed in the revised version of the model [44]). During eccentric contraction, the magnitude of the negative mechanical work rate can exceed that of the total (positive) heat rate, resulting in a net absorption of energy by the fibers. Experiments indicate that the chemical processes involved in fiber contraction cannot be reversed during active lengthening [46], and most of the energy that is absorbed during eccentric contraction (in increased cross-bridge potential energy, for example) is eventually converted into heat [47]. Thus, we prevented the total instantaneous power from becoming negative by immediately dissipating any energy absorbed by the fibers during eccentric contraction (i.e., we constrained the total instantaneous power to be non-negative in our model). Including negative mechanical work and constraining total instantaneous power to be non-negative align with the conclusions of Miller [45].

**Fig 2:**
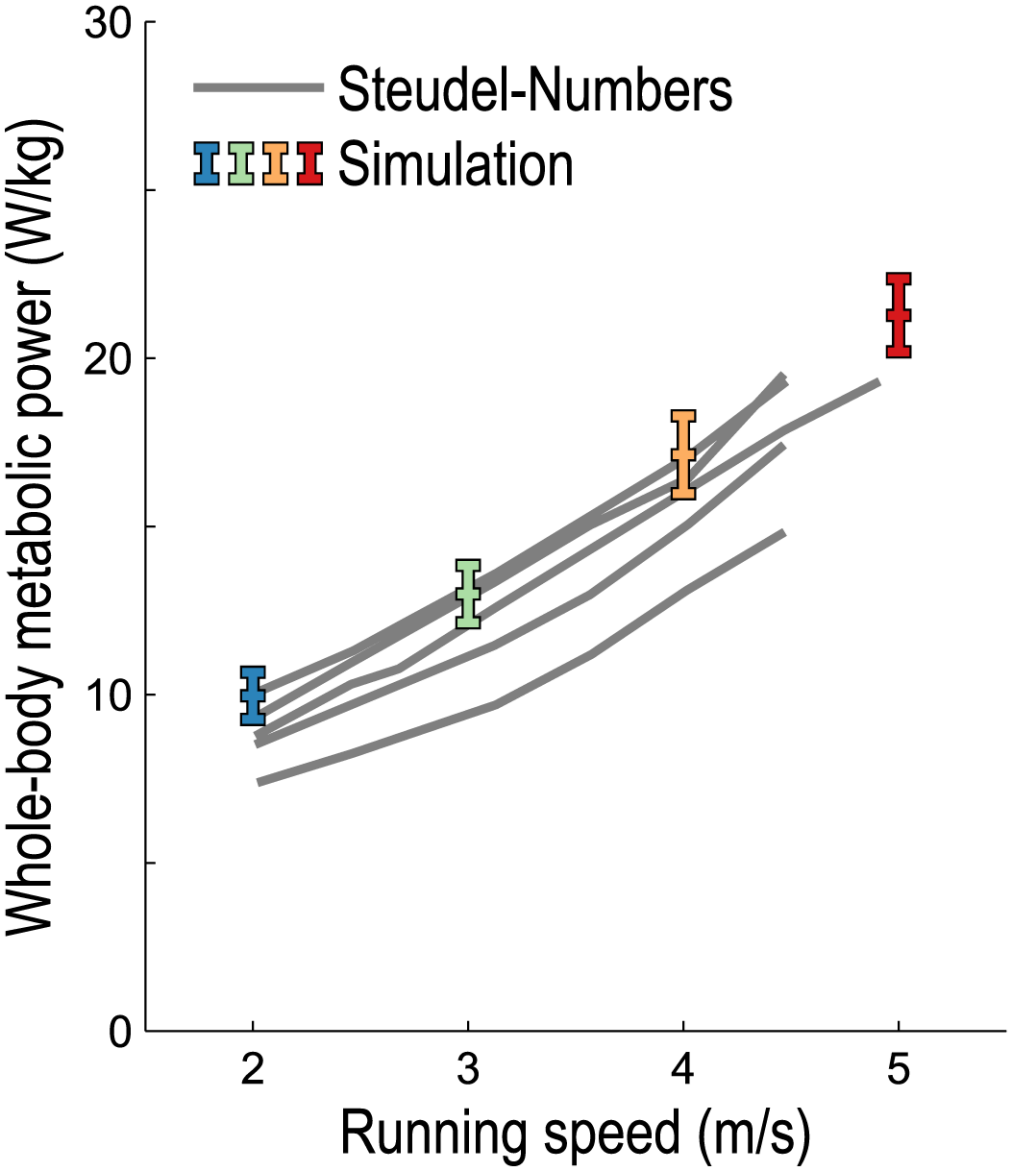
Average whole-body metabolic power consumed during running, normalized by subject mass. The experimental data (gray lines) were obtained by indirect calorimetry collected from 5 males of sufficient fitness to run at all speeds aerobically, as reported by Steudel-Numbers and Wall-Scheffler [48]. Simulations of running at 2, 3, 4, and 5 m/s were generated for 10 male long-distance runners using the methods reported by Hamner and Delp [33] with tendon force–strain curves that fit experimental data (see the curve labeled “E” in Fig 1); colored bars indicate mean ±1 standard deviation. A basal rate of 1.13 W/kg [45, 49] was added to the simulation results to form a valid comparison with the gross metabolic power reported by Steudel-Numbers and Wall-Scheffler.

Incorporating the orderly recruitment model and including negative mechanical work both had the effect of decreasing the metabolic power predicted by our model, while preventing the total instantaneous power from becoming negative had the opposite effect. As shown in Fig 2, our predictions of whole-body metabolic power consumption during running compare favorably with indirect calorimetry data reported by Steudel-Numbers and Wall-Scheffler [48].

### Calculating Power Consumption

We calculated the average power consumed by each muscle during each running gait cycle as follows:

$$\begin{array}{c} P_{\text{avg}}=\frac{m}{t_{1}-t_{0}}\int_{t_{0}}^{t_{1}}\dot{E}\left(t\right)\;dt, \end{array}$$ (5)

where *P*_avg_ is the average power consumed during the gait cycle (from *t*_0_ to *t*_1_), *m* is the muscle mass as computed in Eq (1), and $\dot{E}\left(t\right)$ is the metabolic power consumed, normalized by muscle mass, as predicted by our muscle energetics model. For each muscle, we computed the mean *P*_avg_ over all cycles for each subject at each speed and for each tendon force–strain curve. We then computed the mean and standard deviation across all subjects. Whole-body metabolic power was computed as the sum of the power consumed by all muscles in the model (we added a basal rate of 1.13 W/kg [45, 49] only when comparing our simulation results to experimental measurements of gross metabolic power). The average metabolic power consumption ascribed to a particular joint was calculated in the sagittal plane as follows:

$$\begin{array}{c} P_{\text{joint}}=\sum_{i}P_{\text{uni}}^{\left[i\right]}+\sum_{j}(\frac{m^{\left[j\right]}}{t_{1}-t_{0}}\int_{t_{0}}^{t_{1}}\frac{r_{1}^{\left[j\right]}\left(t\right)}{r_{1}^{\left[j\right]}\left(t\right)+r_{2}^{\left[j\right]}\left(t\right)}{\dot{E}}^{\left[j\right]}\left(t\right)\;dt), \end{array}$$ (6)

where $P_{\text{uni}}^{\left[i\right]}$ is the average power consumed by the *i*th uniarticular muscle crossing the joint of interest, $r_{1}^{\left[j\right]}\left(t\right)\geq 0$ is the instantaneous flexion/extension moment arm of the *j*th biarticular muscle at the joint of interest, and $r_{2}^{\left[j\right]}\left(t\right)\geq 0$ is the analogous moment arm at the other joint crossed by the *j*th biarticular muscle.

## Results

**Fig 3:**
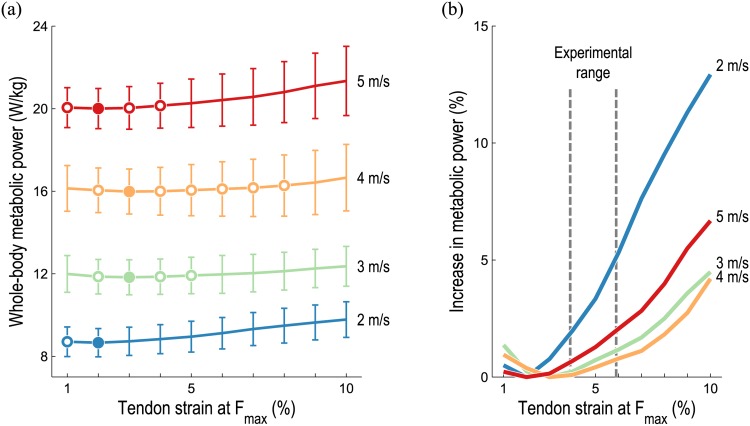
Average whole-body metabolic power consumed during running as tendon compliance varies. The mean (line) and standard deviation (vertical bars) predicted by our simulations at each speed and tendon compliance are shown in (a); the mean increase in average metabolic power from the lowest average power at each speed is shown in (b), expressed as a percentage of the lowest average power at each speed. Filled circles in (a) indicate the lowest value at each speed; open circles denote values that are not significantly greater than these minima (*p* < 0.05, matched pairs t-test). The “experimental range” indicated in (b) is 4.9±1% strain at *F*_max_, the mean and standard deviation reported by Maganaris and Paul [31]. The optimal tendon compliance was near this range for all running speeds; less compliant tendons were substantially more favorable when running at 2 m/s.

Average whole-body metabolic power consumption increased as running speed increased, regardless of tendon compliance, as shown in Fig 3(a). We also found that average whole-body metabolic power was lowest when tendon strain was 2–3% at *F*_max_, which falls below the experimental range of 4.9±1% reported by Maganaris and Paul [31] for the human gastrocnemius tendon, but is near the 3.3% suggested by Zajac [32]. Also note that the average whole-body metabolic power corresponding to higher tendon compliances (as high as 8% strain at *F*_max_) were not significantly greater than these minima. As shown in Fig 3(b), the average whole-body metabolic power at each running speed increased as tendon compliance increased or decreased from the compliance at which the average metabolic power was lowest—that is, average metabolic power consumption was an approximately convex function of tendon compliance at each running speed. The greatest increase in average whole-body metabolic power occurred when running at 2 m/s with very compliant tendons.

**Fig 4:**
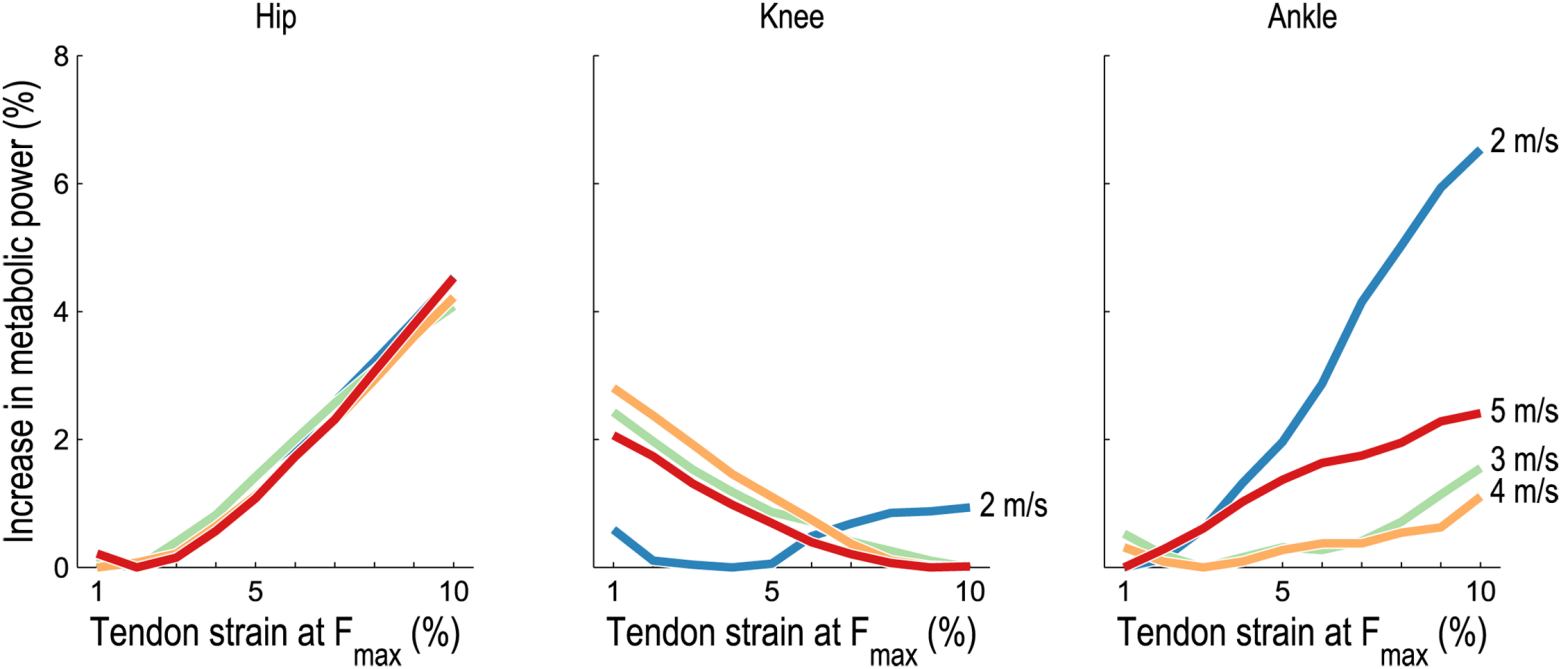
Average metabolic power consumed by lower extremity muscles during running as tendon compliance varies. The mean increase in average metabolic power from the lowest average power at each speed is shown for lower extremity muscles crossing the hip, knee, and ankle, expressed as a percentage of the lowest average whole-body metabolic power at each speed (filled circles in Fig 3(a)). The metabolic power associated with biarticular muscles was distributed between each joint in proportion to instantaneous flexion/extension moment arms. At the knee, more compliant tendons were favored when running at 3–5 m/s. At the ankle, very compliant tendons were particularly detrimental when running at 2 m/s.

Increases in whole-body metabolic power consumption are ultimately due to increases in the power expended by individual muscles; the increases predicted for the muscles crossing the hip, knee, and ankle are shown in Fig 4. As was the case for the whole body, average metabolic power consumption was an approximately convex function of tendon compliance at each running speed for each joint. The percent increase in average metabolic power associated with muscles crossing the hip was similar at all speeds. The average metabolic power associated with muscles crossing the knee was lowest at low tendon compliance when running at 2 m/s; however, when running at 3–5 m/s, the average metabolic power was lowest at high tendon compliance. Finally, muscles crossing the ankle generally exhibited an increase in average metabolic power with increasing tendon compliance at all speeds, with the greatest increase occurring when running at 2 m/s.

**Fig 5:**
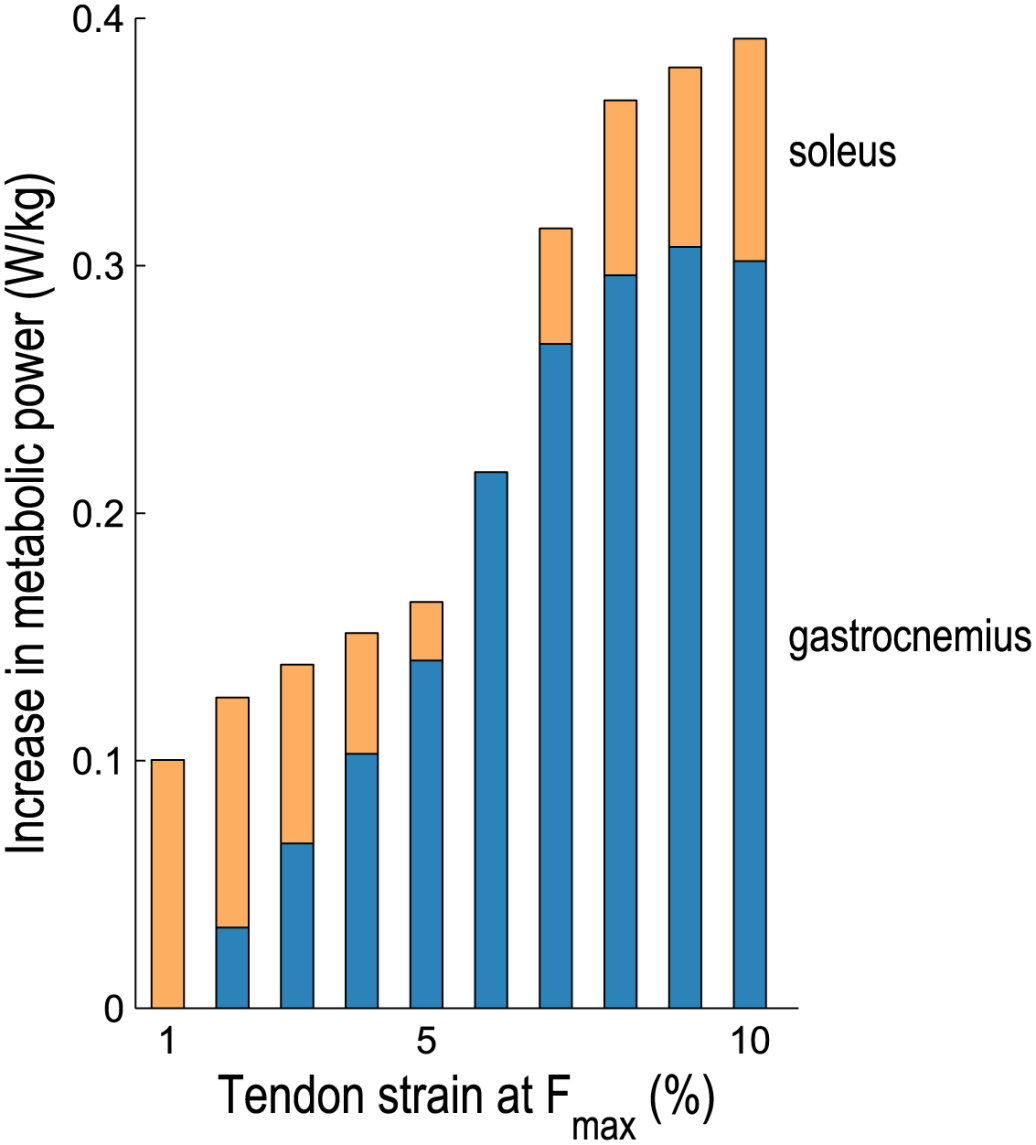
Average metabolic power consumed by the gastrocnemius and soleus muscles during running as tendon compliance varies. The mean increase in average metabolic power from the lowest average power consumed is shown when running at 2 m/s. The gastrocnemius (medial and lateral heads) consumed the greatest power when tendons were very compliant, as did all the other plantarflexors except the soleus, which consumed the greatest power when tendons were least compliant.

**Fig 6:**
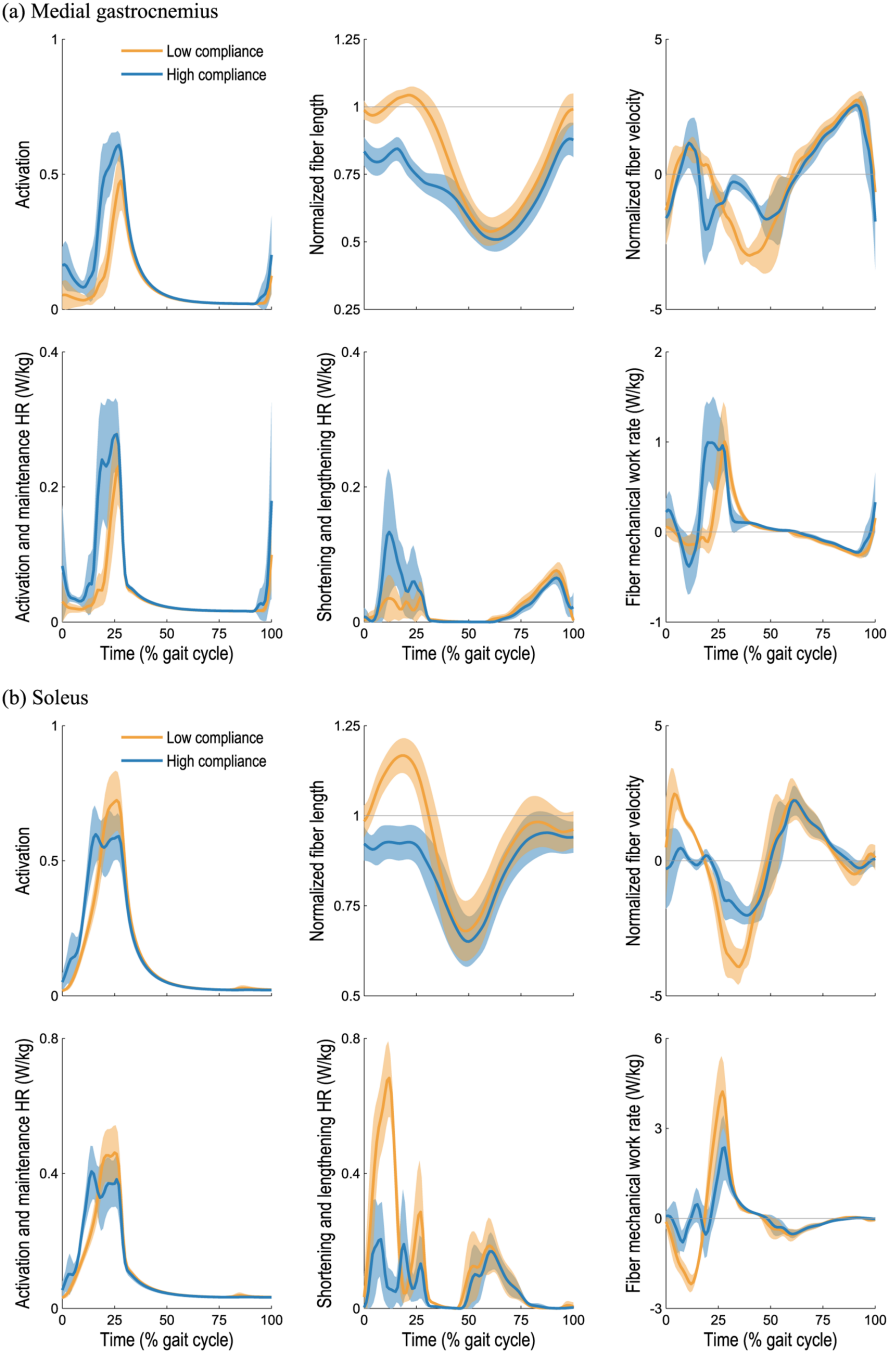
Dynamics and metabolics of the medial gastrocnemius and soleus muscles during running at 2 m/s. Simulated muscle activations, fiber lengths, and fiber velocities (top row) and outputs from our model of muscle energetics (bottom row) for the right medial gastrocnemius (a) and soleus (b) muscles are shown over the gait cycle. Our model of muscle energetics predicted the rate of heat generation due to sarcoplasmic reticular ion transport and actin–myosin interaction (activation and maintenance heat rate), the rate of heat generation due to shortening and lengthening of the fibers, and the mechanical power of the fibers [37]. The mean (line) and standard deviation (shaded region) are shown for the seven rearfoot-striking subjects when low (2% strain at *F*_max_; orange) and high (10% strain at *F*_max_; blue) tendon compliances were used. When tendons were very compliant, the soleus fibers were operating nearly isometrically during stance, thereby reducing the average shortening and lengthening heat rate predicted by the energetics model (from 128 to 61 mW/kg). In contrast, the medial gastrocnemius fibers were operating far from their optimal lengths during stance when tendons were very compliant, thereby requiring greater activation to generate a similar plantarflexion moment and increasing the average activation and maintenance heat rate (from 38 to 57 mW/kg).

Of the muscles crossing the ankle, the gastrocnemius was the largest contributor to the increase in average metabolic power with tendon compliance when running at 2 m/s, as shown in Fig 5. An analysis of the dynamics and metabolics of the medial gastrocnemius muscle is shown in Fig 6(a). At low tendon compliance, the medial gastrocnemius was operating close to its optimal fiber length during stance (0–47% gait cycle), thereby requiring a relatively small activation and consuming a relatively small amount of metabolic power. In contrast, when its tendon was very compliant, the medial gastrocnemius muscle fibers were shorter and operating far from their optimal lengths during stance, requiring greater activation to generate a similar ankle plantarflexion moment. Consequently, the activation and maintenance heat rate was greater when the tendon was very compliant. The fiber velocity was also higher for much of the stance phase when the tendon was very compliant, thereby increasing both the shortening and lengthening heat rate and the mechanical work rate. The medial gastrocnemius muscle generated relatively small forces and consumed relatively little energy during the swing phase, thus tendon compliance had little effect on energy consumption during swing.

The soleus was the only plantarflexor muscle that consumed the greatest metabolic power at low tendon compliance; all other plantarflexors experienced the greatest increase in average metabolic power at high tendon compliance (see Fig 5). An analysis of the dynamics and metabolics of the soleus muscle is shown in Fig 6(b). When its tendon was very compliant, the soleus was operating nearly isometrically during stance; a small fiber velocity at this critical force-generating phase of gait translated into a small shortening and lengthening heat rate and a small mechanical work rate. The integral of the squared activation was nearly the same regardless of tendon compliance, as was the activation and maintenance heat rate as a result. Once again, the muscle generated relatively small forces and consumed relatively little energy during the swing phase, regardless of tendon compliance.

**Fig 7:**
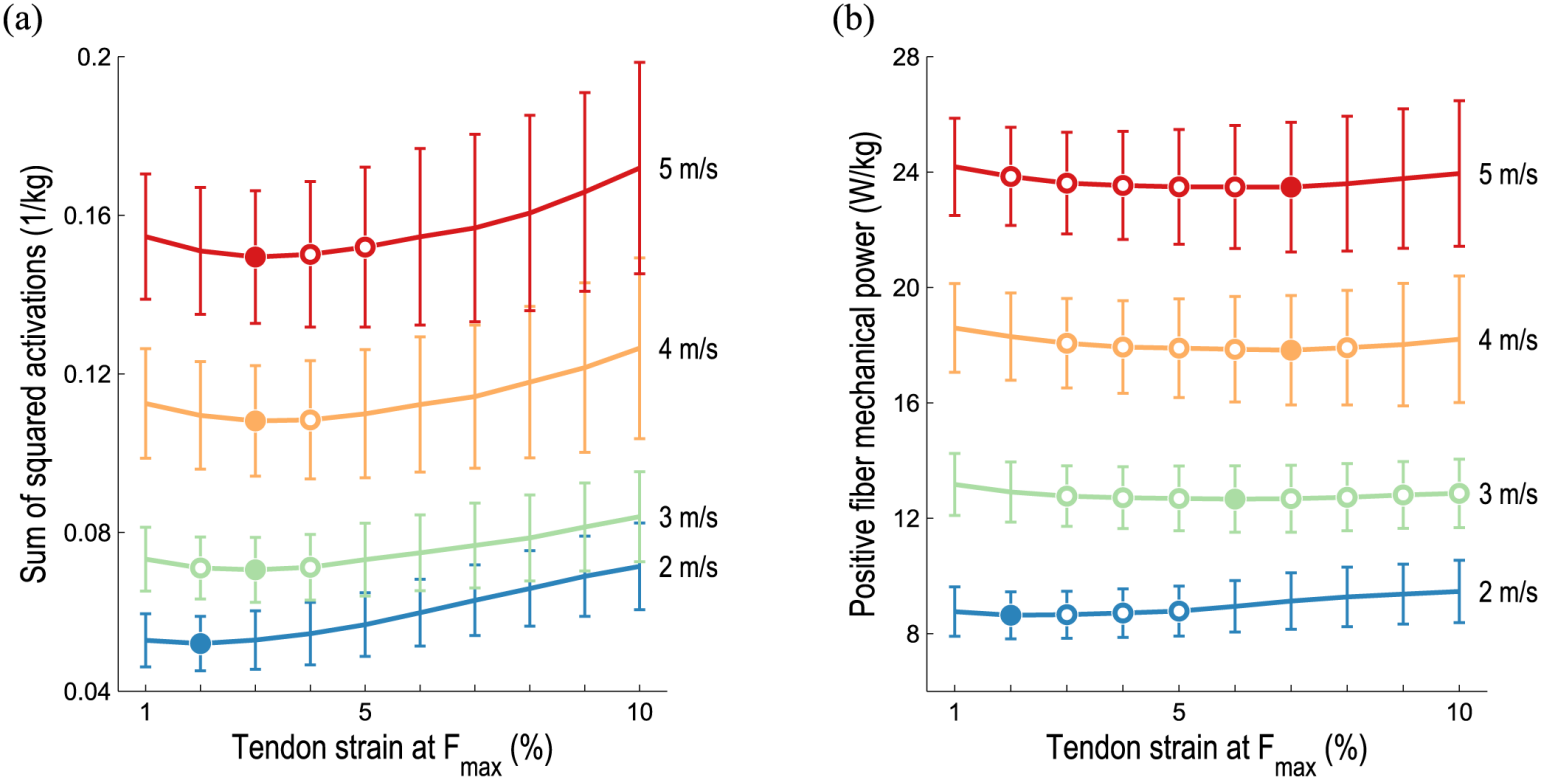
Muscle activations and positive fiber mechanical power during running as tendon compliance varies. The mean (line) and standard deviation (vertical bars) at each speed and tendon compliance are shown. Filled circles indicate the lowest value at each speed; open circles denote values that are not significantly greater than these minima (*p* < 0.05, matched pairs t-test). Although the sum of squared activations (a) reveals similar trends as average metabolic power at the whole-body level, activation-based metrics may disagree with metabolics at the muscle level (e.g., see Fig 6(b)). Comparison of Figs 3(a) and 7(b) reveals that average positive fiber mechanical power achieved minima at greater tendon compliances than average whole-body metabolic power consumption, suggesting that positive fiber mechanical power is a poor surrogate for metabolic power.

At the whole-body level, the sum of squared muscle activations exhibited trends similar to those of average metabolic power consumption as tendon compliance varied, as shown in Fig 7(a). The lowest sum of squared muscle activations occurred for all running speeds when tendon strain was 2–3% at *F*_max_. In contrast, the sum of the average positive fiber mechanical power of all muscles was lowest when tendon strain was 2–7% at *F*_max_, as shown in Fig 7(b). This metric was less sensitive to tendon compliance than both average whole-body metabolic power consumption (Fig 3(a)) and the sum of squared muscle activations (Fig 7(a)).

## Discussion

The results of this study suggest that tendon compliances of 1–10% strain at *F*_max_ can change the average whole-body metabolic power by up to 1.1 W/kg when running at 2 m/s, which is about 13% of the lowest average whole-body metabolic power at this speed; smaller (but statistically significant) increases of approximately 4–7% were predicted when running at 3–5 m/s with very compliant tendons. These effects are of a similar magnitude as the 6–8% reductions in whole-body metabolic power achieved by the state-of-the-art assistive devices for unloaded and loaded walking [50–52], as well as the 3–5% increase in whole-body energy consumption observed when running at 2.7 m/s with 3.6 kg (approximately 5% bodyweight) attached to the waist [53]. Thus, the effects we observed may be considered substantial.

We sought to explore three hypotheses, the first being that whole-body metabolic power consumption is a convex function of tendon compliance, reaching its lowest value when tendon strain is approximately 3–4% at *F*_max_, the range of tendon stretch suggested by Zajac [32]. Our simulations support this hypothesis, as illustrated in Fig 3(a). Average whole-body metabolic power consumption was found to be an approximately convex function of tendon compliance at all running speeds. Although the lowest average whole-body metabolic power occurred when tendon strain was 2–3% at *F*_max_, which is slightly below the values reported by Zajac and others [30–32], the metabolic cost corresponding to higher tendon compliances (e.g., up to 5% strain at *F*_max_ when running at 3 m/s) were not significantly greater than these minima. Interestingly, the optimal tendon compliance was similar at all speeds. This result supports the principle that humans naturally adjust their gait to minimize energy consumption; the subjects in the present study would have naturally selected the stride frequencies necessary to retain optimal spring-like behavior [8, 54, 55]. Because the properties of human tendons do not vary as we change our running speed, one might expect the predicted tendon compliance that minimizes whole-body metabolic power to be relatively insensitive to running speed.

Our second hypothesis was that, for very low and very high tendon compliances, the muscles crossing the ankle experience a greater increase in metabolic power than those crossing the knee or hip. As shown in Fig 4, this hypothesis appears to be true only when running at 2 m/s; at higher running speeds, the muscles crossing the hip experienced the greatest increase in metabolic cost with increasing tendon compliance. These results appear to coincide with the known shift in power generation from the ankle to the hip as running speed increases [5]. Thus, at higher speeds, the dynamics of the muscles crossing the hip may play an increasingly important role in the economy of running.

Our simulations suggest the existence of two competing behaviors in the triceps surae muscle group: one that results in greater economy at high tendon compliance and one that results in greater economy at low tendon compliance. As shown in Fig 6(b), the soleus muscle consumed less metabolic power at high tendon compliance because, in this case, its fibers operated close to their optimal lengths and nearly isometrically during stance. A lower fiber velocity resulted in a lower shortening and lengthening heat rate as well as a lower mechanical work rate, both of which reduced the metabolic power consumption predicted by our muscle energetics model. The fiber lengths we obtained at high tendon compliance are in close agreement with those reported elsewhere for running at similar speeds [16, 24, 56, 57]. Our results at high tendon compliance support several conclusions made by Rubenson et al. [24] for running at 3 m/s: the soleus fibers are nearly isometric during mid-stance, shorten when recruited during late stance and toe-off, and operate on the ascending limb of the active force–length curve throughout the gait cycle, where the muscle length remains stable when perturbed [58]. The study of Hof et al. [18] indicates that the soleus fibers do little negative work, further supporting the result we obtained at high tendon compliance.

Unlike the soleus muscle, the medial gastrocnemius consumed more metabolic power at high tendon compliance, as shown in Fig 6(a). When the medial gastrocnemius tendon was very compliant, it was primarily the tendon (not the muscle fibers) that stretched during knee extension and ankle dorsiflexion prior to heel-strike, which stored energy in the calcaneal tendon but resulted in relatively short muscle fibers that were far from their optimal lengths during stance. At low tendon compliance, on the other hand, the fibers stretched to their optimal lengths prior to heel-strike and the muscle was able to generate the necessary plantarflexion moment with lower activation. Consequently, the activation and maintenance heat rate term in the metabolic model was lower at low tendon compliance. Lichtwark et al. [14] used ultrasonography to examine the medial gastrocnemius during running at 2 m/s and found that the fibers were shortening throughout stance; their observed fiber length trajectories correspond roughly to those predicted by our simulations at high tendon compliance (i.e., 10% strain at *F*_max_) and to those reported elsewhere [16, 21]. More recent experiments using ultrasonography have reported tendon strains exceeding 7% in the medial gastrocnemius during a maximal voluntary contraction [59], which does not include the substantial strain of the aponeurosis [60]. The in vivo experiments of Hoang et al. [61] on relaxed muscle suggest that the gastrocnemius tendon and aponeurosis undergo strains of 9.2±4.1% across the physiological range of motion. In our simulations, the soleus muscle consumed an average of 28 mW/kg less metabolic power at high tendon compliance during running at 2 m/s, while the medial gastrocnemius experienced an increase of 93 mW/kg when its tendon was very compliant.

There are several explanations for why the soleus and gastrocnemius fiber lengths reported elsewhere are in closest agreement with the fiber lengths we obtained at high tendon compliance. Perhaps most importantly, calcaneal tendon compliance varies substantially between individuals [62, 63]. Tendon compliance can be affected by resistance and strength training [64] and by fatiguing repetitive conditions such as those experienced in running [65]. Studies have shown that the most economical distance runners have less compliant calcaneal tendons than average [65, 66], which is suggested by Fig 5—though efficient walking requires more compliant tendons than efficient running [62], indicating a trade-off between walking and running economy. This evidence suggests that it may be prudent to tune calcaneal tendon compliance in subject-specific models. Arnold et al. [16] used a musculoskeletal model similar to that used in the present study [36] to perform electromyography-driven simulations of walking and running, but modified the compliance of the calcaneal tendon to obtain ankle moment and power results that were in agreement with those of an inverse dynamics analysis. In fact, Arnold et al. used a tendon force–strain curve that produced 10% strain at *F*_max_ for the soleus, medial gastrocnemius, and lateral gastrocnemius musculotendon actuators. The fiber lengths obtained in our simulations appear to support this choice of tendon compliance for our subjects (though see the Study Limitations section, below).

Our third hypothesis was that metrics based solely on muscle activations or positive fiber mechanical power exhibit trends that are different from the predictions of a detailed muscle energetics model. At the whole-body level, our simulations predicted similar trends between average metabolic power (Fig 3(a)) and the sum of squared muscle activations (Fig 7(a)), supporting the use of activation-based metrics when minimizing energy consumption [34, 67]. At the muscle level, however, an activation-based metric may be misleading. For example, note that the integrals of the squared activations shown in Fig 6(b) are nearly identical, despite the fact that the soleus consumes considerably less metabolic power when its tendon is compliant (due to the reduced shortening and lengthening heat rate term). We also note that our muscle energetics model provides a prediction of energy expenditure in meaningful physical units, which can be directly related to caloric consumption or power requirements in an assistive device, for example. Different trends were observed between average whole-body metabolic power (Fig 3(a)) and average positive fiber mechanical power (Fig 7(b)), suggesting that the latter—though simpler to compute—is a poor surrogate for metabolic cost.

### Study Limitations

There are several limitations of this study. First, the compliances of all tendons in the model were adjusted simultaneously—that is, we assumed that all tendons have the same stress–strain curve. Comparisons of in vivo measurements of the human gastrocnemius and tibialis anterior tendons suggest that the material properties of tendon are similar in muscles of different function [31]. Also note, however, that the in vivo measurements of Franz et al. [68] indicate that the superficial and deep regions of the calcaneal tendon undergo different amounts of deformation during walking, suggesting that the soleus and gastrocnemius muscles may be best represented in simulation with tendons of different compliance. In fact, the sliding between adjacent tendon fascicles may play an important role in the storage and release of elastic energy [69], but these dynamics have not been fully characterized and, thus, are not modeled in our simulations.

A second limitation of this study relates to the uncertainty associated with the parameters in our musculoskeletal model—particularly the tendon slack lengths, to which the model is known to be especially sensitive [70–72]. Unfortunately, a direct measurement of tendon slack length is difficult to obtain due to the aponeurosis and possible changes in tendon length post-mortem [72]. Thus, although our results support the use of a tendon force–strain curve that produces 10% strain at *F*_max_ for the soleus and gastrocnemii, as was done by Arnold et al. [16], increasing the tendon slack length would have a similar effect on the lengths of these fibers. The tendon slack lengths in our model were determined based on in vivo measurements of passive and active joint moments [73]; validating these lengths experimentally (e.g., with in vivo sarcomere length measurements [74]) would greatly enhance the reliability of our predictions.

A third limitation involves use of the Computed Muscle Control (CMC) algorithm to generate muscle-driven simulations. CMC solves the muscle redundancy problem by minimizing the sum of squared muscle activations at individual time steps of a simulation. Although this objective is physiologically based [75] and has been shown to generate realistic kinematics in predictive simulations [76], it may be more appropriate to consider a longer CMC time window [34], or to minimize energy consumption [77] or fatigue [78]. Indeed, as suggested above, activations alone are not sufficiently descriptive to distinguish between metabolically beneficial and detrimental scenarios at the muscular level. Additional criteria not considered here include maintaining balance, avoiding injury, stabilizing joints, reducing fatigue, and maintaining force-generating capacity, each of which could cause tendon compliance to differ from the compliance at which metabolic cost is lowest. In using CMC, we also assumed that the kinematics and ground reaction forces observed experimentally would not change as tendon compliance varied. This assumption is supported, in part, by the work of Hof et al. [18], who observed similar ankle kinematics in subjects with high and low calcaneal tendon compliances.

A fourth limitation is the set of simplifications involved in modeling the human musculoskeletal system. The musculotendon model used in this study [79, 80] ignores the role of past states [81], temperature [82], and fatigue [83], which may have important metabolic consequences. Our model of musculotendon dynamics also ignores the heterogeneity of muscle and tendon fibers [69] and the hysteresis evident in experiments of tendon loading and unloading [31]. Furthermore, the soleus and gastrocnemius muscles were modeled with separate tendons rather than a single shared calcaneal tendon, the consequence of which is unknown—though the fiber lengths we obtained with high tendon compliance compared favorably with those reported elsewhere [14, 16, 21, 24, 56, 57]. Also note that we use a muscle model consisting of a single representative fiber rather than many fibers of different lengths, stretching at different speeds, and having different moment arms about the joints they span. This approximation ultimately leads to an exaggeration of the reduction in muscle force-generation capacity due to fiber kinematics (i.e., the length and velocity of the representative fiber). Use of more detailed models of musculotendon dynamics [84] may alleviate our current need to use high specific tensions in our models. Finally, we ignored the energy expended by the upper body muscles, which would introduce a relatively small offset in average whole-body metabolic power consumption and would have little effect on the metabolic cost increases we studied.

### Conclusions

Despite these limitations, we believe two important conclusions can be drawn from this study. First, more compliant tendons are not always metabolically advantageous. In particular, our simulations predict that the soleus muscle benefits metabolically from a very compliant tendon when running, but that the gastrocnemii do not. Musculoskeletal modeling and simulation would benefit from further experimentation on the triceps surae muscle group, particularly investigations involving both ultrasonography and direct measurements of sarcomere lengths [85] to support model calibration and validation, especially of tendon slack length. Our second conclusion is that our model of muscle energy expenditure provides deeper insight than simpler metrics based solely on muscle activations or positive fiber mechanical power. Muscle energetics models enable examination of energy consumption with unprecedented detail, complementing indirect calorimetry measurements obtained experimentally at the whole-body level. Our muscle energetics model is available in OpenSim 3.3, an open-source software platform for modeling and simulation of movement.
